# Supplementary material for: An investigation into gender distributions in scholarly publications among dental faculty members in Iran
Source: PLoS One. 2024 Jun 27;19(6):e0300698. doi: 10.1371/journal.pone.0300698 (PMC11210791; doi:10.1371/journal.pone.0300698)
Supplement: S1 Table — (DOCX) [file pone.0300698.s001.docx]

**Gender inequality in each speciality**

**Number of dental faculty members**

Oral and maxillofacial surgery (OMFS) (N men=131) and prosthodontics (N men=135) had the highest MtoW ratio (5.5 and 1.1, respectively) whereas pediatric dentistry (N women=163, MtoW ratio=0.2), restorative dentistry (N women=163, MtoW ratio=0.3), oral medicine (N women=138, MtoW ratio=0.3), and pathology (N women=69, MtoW ratio=0.3) had the lowest MtoW ratio. Full details are available in Supplementary Table 1.

Supplementary Table 1. Number of dental faculty members in each speciality (*: ratio > 1)

| Speciality | Men | Women | MtoW |
| --- | --- | --- | --- |
| COH | 11 | 25 | 0.4 |
| Dental Materials | 3 | 7 | 0.4 |
| Endodontics | 110 | 119 | 0.9 |
| OMFS | 131 | 24 | **5.5*** |
| Oral Medicine | 35 | 138 | 0.3 |
| Orthodontics | 96 | 97 | 1.0 |
| Pathology | 22 | 69 | 0.3 |
| Pediatric Dentistry | 33 | 163 | 0.2 |
| Periodontics | 81 | 96 | 0.8 |
| Prosthodontics | 135 | 118 | **1.1*** |
| Radiology | 39 | 86 | 0.5 |
| Restorative Dentistry | 53 | 163 | 0.3 |

MtoW: Men-to-Women ratio; COH: Community Oral Health; OMFS: Oral and Maxillofacial Surgery; Pathology: Oral and Maxillofacial Pathology; Radiology: Oral and Maxillofacial Radiology.
