# Supplementary material for: An investigation into gender distributions in scholarly publications among dental faculty members in Iran
Source: PLoS One. 2024 Jun 27;19(6):e0300698. doi: 10.1371/journal.pone.0300698 (PMC11210791; doi:10.1371/journal.pone.0300698)
Supplement: S2 Table — (DOCX) [file pone.0300698.s002.docx]

**Gender inequality in each speciality**

**h-index**

Restorative dentistry and paediatric dentistry had the highest MtoW ratio (1.5 for both). In contrast, dental materials and OMFS had the lowest ratios (0.42 and 0.5, respectively). Women and men in dental materials had the highest median of H-index (12 (IQR=10.5) and 5 (IQR=0.5), respectively). The median of H-index was one for faculty members in both prosthodontics and restorative dentistry. Full details are available in Supplementary Table 2.

Supplementary Table 2. Dental faculty members’ h-index by gender and speciality (SD) (*: lower than 1)

| Speciality | Median (IQR) | | | MtoW |
| --- | --- | --- | --- | --- |
|  | Both sexes | Men | Women |  |
| COH | 3 (3.25) | 4 (2.5) | 3 (3) | 1.33 |
| Dental Materials | 5 (8.75) | 5 (0.5) | 12 (10.5) | 0.42* |
| Endodontics | 2 (4) | 3 (5.8) | 2 (4) | 1.5 |
| OMFS | 1 (3) | 1 (4) | 2 (2.5) | 0.5* |
| Oral Medicine | 2 (3) | 2 (2.5) | 2 (3) | 1 |
| Orthodontics | 1 (3) | 2 (3) | 1 (2) | 2 |
| Pathology | 2 (4) | 3 (4) | 2 (4) | 1.5 |
| Pediatric Dentistry | 1 (2) | 2 (3.25) | 1 (2) | 2 |
| Periodontics | 1 (3) | 2 (4) | 1 (3) | 2 |
| Prosthodontics | 1 (2) | 1 (2) | 1 (2) | 1 |
| Radiology | 1 (3) | 1.5 (3) | 1 (3) | 1.5 |
| Restorative Dentistry | 1 (2.25) | 1 (4) | 1 (2) | 1 |

IQR: Inter-Quartile Range; MtoW: Men-to-Women ratio; COH: Community Oral Health; OMFS: Oral and Maxillofacial Surgery; Pathology: Oral and Maxillofacial Pathology; Radiology: Oral and Maxillofacial Radiology.
