# Supplementary material for: An investigation into gender distributions in scholarly publications among dental faculty members in Iran
Source: PLoS One. 2024 Jun 27;19(6):e0300698. doi: 10.1371/journal.pone.0300698 (PMC11210791; doi:10.1371/journal.pone.0300698)
Supplement: S3 Table — (DOCX) [file pone.0300698.s003.docx]

**Gender inequality in each speciality**

**Number of papers**

Restorative dentistry and paediatric dentistry had the highest MtoW ratio (3 and 2.5, respectively). In contrast, dental material and OMFS showed the lowest ratio (0.37 and 0.67, respectively). Dental materials speciality had the highest median for the number of papers among all specialities (27 (IQR=32) and 10 (IQR=1.5), respectively) whereas women in periodontics (1, IQR=3.75) and restorative dentistry (1, IQR=4) had the lowest mean for the number of papers. Full details are available in Supplementary Table 3.

Supplementary Table 3. Number of papers by gender and speciality (*: lower than 1)

| Speciality | Median (IQR) | | | MtoW |
| --- | --- | --- | --- | --- |
|  | Both | Men | Women |  |
| COH | 4 (6.75) | 7 (12) | 3 (6) | 2.33 |
| Dental Materials | 10.5 (28) | 10 (1.5) | 27 (32) | 0.37* |
| Endodontics | 4 (11) | 7 (15.75) | 3 (8) | 2.33 |
| OMFS | 2 (7) | 2 (7.25) | 3 (5) | 0.67* |
| Oral Medicine | 5 (9) | 5 (8.5) | 5 (9.75) | 1 |
| Orthodontics | 3 (7) | 4 (9.25) | 2 (6) | 2 |
| Pathology | 6 (15) | 6 (18) | 5.5 (15) | 1.09 |
| Pediatric Dentistry | 2 (4) | 5 (7.75) | 2 (3) | 2.5 |
| Periodontics | 2 (9) | 4 (11) | 2 (4) | 2 |
| Prosthodontics | 2 (5) | 2 (5.5) | 1 (3.75) | 2 |
| Radiology | 3 (6.25) | 3 (8.75) | 2.5 (5.75) | 1.2 |
| Restorative Dentistry | 1 (6) | 3 (11.25) | 1 (4) | 3 |

IQR: Inter-Quartile Range; MtoW: Men-to-Women ratio; COH: Community Oral Health; OMFS: Oral and Maxillofacial Surgery; Pathology: Oral and Maxillofacial Pathology; Radiology: Oral and Maxillofacial Radiology.
