# Supplementary material for: An investigation into gender distributions in scholarly publications among dental faculty members in Iran
Source: PLoS One. 2024 Jun 27;19(6):e0300698. doi: 10.1371/journal.pone.0300698 (PMC11210791; doi:10.1371/journal.pone.0300698)
Supplement: S4 Table — (DOCX) [file pone.0300698.s004.docx]

**Gender inequality in each speciality**

**Number of citations per paper**

Restorative dentistry and endodontics had the highest MtoW ratio (2.47 and 1.79, respectively), while dental materials and prosthodontics had the lowest ratio (0.54 and 1.09, respectively). Women and men in dental materials had the highest median for the number of citations per paper among all specialities (17.16 (IQR=10.255) and 9.27 (IQR=7.175), respectively). In contrast, women in orthodontics (2.22, IQR=4.265) and women in oral and maxillofacial radiology (from now on: radiology) (2.715, IQR=3.8275) had the lowest median. Full details are available in Supplementary Table 4.

Supplementary Table 4. Citations per papers by gender and speciality (*: lower than 1)

| Speciality | Median (IQR) | | | MtoW |
| --- | --- | --- | --- | --- |
|  | Both | Men | Women |  |
| COH | 6 (6.4) | 6.93 (2.465) | 4.57 (7.355) | 1.52 |
| Dental Materials | 13.2 (9.46) | 9.27 (7.175) | 17.16 (10.255) | 0.54* |
| Endodontics | 5.88 (7.74) | 7.395 (8.655) | 4.14 (7) | 1.79 |
| OMFS | 4.58 (5.67) | 4.75 (6.11) | 3.6 (3.9) | 1.32 |
| Oral Medicine | 3.6 (4.22) | 4.67 (4.625) | 3.5 (4.285) | 1.33 |
| Orthodontics | 3 (5.04) | 3.475 (5.2075) | 2.22 (4.265) | 1.57 |
| Pathology | 3.86 (5.20) | 5.9 (4.6) | 3.67 (5.18) | 1.61 |
| Pediatric Dentistry | 3.29 (5) | 3.93 (5.905) | 3 (4.28) | 1.31 |
| Periodontics | 5 (6) | 5.41 (5.96) | 4.5 (6.305) | 1.20 |
| Prosthodontics | 3 (5) | 3 (4.67) | 2.75 (4.125) | 1.09 |
| Radiology | 3 (4.38) | 4 (3.4325) | 2.715 (3.8275) | 1.47 |
| Restorative Dentistry | 4 (7) | 6.89 (5.555) | 2.79 (6) | 2.47 |

IQR: Inter-Quartile Range; MtoW: Men-to-Women ratio; COH: Community Oral Health; OMFS: Oral and Maxillofacial Surgery; Pathology: Oral and Maxillofacial Pathology; Radiology: Oral and Maxillofacial Radiology.
