# Supplementary material for: An investigation into gender distributions in scholarly publications among dental faculty members in Iran
Source: PLoS One. 2024 Jun 27;19(6):e0300698. doi: 10.1371/journal.pone.0300698 (PMC11210791; doi:10.1371/journal.pone.0300698)
Supplement: S5 Table — (DOCX) [file pone.0300698.s005.docx]

**Gender inequality in each speciality**

**First-author papers percentage**

Periodontics and OMFS had the highest MtoW ratio (2.54 and 2.50, respectively). In contrast, pathology and COH had the lowest ratio (0.70 and 0.79, respectively). Women and men in COH had the highest median for first author percentage (63.07 (IQR=44.35) and 50 (IQR=36.55), respectively), whereas men and women in radiology and women in paediatric dentistry had the lowest median (0). Full details are available in Supplementary Table 5.

Supplementary Tabel 5. The percentage of first-author papers by gender and speciality (*: ratio < 1, ^: ratio > 2)

| Speciality | Median (IQR) | | | MtoW |
| --- | --- | --- | --- | --- |
|  | Both | Men | Women |  |
| COH | 57.14 (42.86) | 50 (36.55) | 63.07 (44.35) | 0.79 |
| Dental Materials | 27.33 (22.16) | 37.5 (22.73) | 22.22 (17.79) | 1.69 |
| Endodontics | 24.57 (44.27) | 25 (30.79) | 20.71 (50) | 1.21 |
| OMFS | 16.67 (50) | 16.67 (44.44) | 6.67 (50) | 2.50 |
| Oral Medicine | 25 (48.81) | 33.33 (28.08) | 25 (48.21) | 1.33 |
| Orthodontics | 12.5 (50) | 13.39 (45.71) | 10 (50) | 1.34 |
| Pathology | 22.5 (50) | 20 (42.83) | 28.57 (50) | 0.70 |
| Pediatric Dentistry | 11.11 (50) | 29.62 (50) | 0 (50) | - |
| Periodontics | 25 (41.43) | 28.57 (46.67) | 11.25 (39.38) | 2.54 |
| Prosthodontics | 18.55 (45.95) | 19.44 (50) | 14.29 (43.94) | 1.36 |
| Radiology | 0 (44.64) | 0 (33.57) | 0 (50) | - |
| Restorative Dentistry | 14.58 (46.15) | 25 (43.32) | 11.11 (50) | 2.25 |

IQR: Inter-Quartile Range; MtoW: Men-to-Women ratio; COH: Community Oral Health; OMFS: Oral and Maxillofacial Surgery; Pathology: Oral and Maxillofacial Pathology; Radiology: Oral and Maxillofacial Radiology.
