# Supplementary material for: An investigation into gender distributions in scholarly publications among dental faculty members in Iran
Source: PLoS One. 2024 Jun 27;19(6):e0300698. doi: 10.1371/journal.pone.0300698 (PMC11210791; doi:10.1371/journal.pone.0300698)
Supplement: S6 Table — (DOCX) [file pone.0300698.s006.docx]

**Gender inequality in each speciality**

### Corresponding-author percentage

Dental materials and COH had the highest MtoW ratio (both 1.13), while radiology and restorative dentistry had the lowest (both 0). Community oral health showed the highest median of corresponding-author paper percentage (35 (IQR=37.7) and 30.95 (IQR=50), respectively). In contrast, men in radiology and restorative dentistry had the lowest median (0). Full details are available in Supplementary Table 6.

Supplementary Tabel 6. The percentage of corresponding-author papers by gender and speciality (*: ratio < 1, ^: ratio > 2)

| Speciality | Median (IQR) | | | MtoW |
| --- | --- | --- | --- | --- |
|  | Both | Men | Women |  |
| COH | 33.33 (57.14) | 35 (37.7) | 30.95 (50) | 1.13 |
| Dental Materials | 23.61 (21.5) | 25 (8.64) | 22.22 (31.46) | 1.13 |
| Endodontics | 21.83 (47.03) | 14.84 (33.33) | 29.81 (45.31) | 0.50 |
| OMFS | 10.24 (40) | 8.7 (41.18) | 12.5 (33.33) | 0.70 |
| Oral Medicine | 23.81 (50) | 23.81 (38.18) | 24.26 (50) | 0.98 |
| Orthodontics | 25 (50) | 23.3 (42.56) | 25 (52.78) | 0.93 |
| Pathology | 32.13 (50) | 10 (34.29) | 33.33 (56.25) | 0.30 |
| Pediatric Dentistry | 25 (50) | 16.23 (46.15) | 31.58 (50) | 0.51 |
| Periodontics | 23.08 (50) | 20 (50) | 25 (50) | 0.8 |
| Prosthodontics | 17.42 (50) | 12.5 (50) | 25 (50) | 0.5 |
| Radiology | 14.29 (50) | 0 (25.57) | 20 (50) | 0 |
| Restorative Dentistry | 16.03 (50) | 0 (20) | 25 (50) | 0 |

IQR: Inter-Quartile Range; MtoW: Men-to-Women ratio; COH: Community Oral Health; OMFS: Oral and Maxillofacial Surgery; Pathology: Oral and Maxillofacial Pathology; Radiology: Oral and Maxillofacial Radiology.
