# Supplementary material for: An investigation into gender distributions in scholarly publications among dental faculty members in Iran
Source: PLoS One. 2024 Jun 27;19(6):e0300698. doi: 10.1371/journal.pone.0300698 (PMC11210791; doi:10.1371/journal.pone.0300698)
Supplement: S7 Table — (DOCX) [file pone.0300698.s007.docx]

**Gender inequality in each speciality**

### Number of papers per year

Periodontics and paediatric dentistry had the highest MtoW ratio (1.93 and 1.91, respectively). In contrast, dental materials and OMFS had the lowest ratio (0.49 and 0.70, respectively). Dental materials had the highest median for the number of papers per year (1.69 (IQR=1.01) and 0.83 (IQR=0.21), respectively) and women in restorative dentistry (0.28, IQR=0.67) and periodontics (0.3, IQR=0.75) had the lowest mean. Full details are available in Supplementary Table 7.

Supplementary Tabel 7. Number of papers per year by gender and speciality

| Speciality | Median (IQR) | | | MtoW |
| --- | --- | --- | --- | --- |
|  | Both | Men | Women |  |
| COH | 0.55 (0.61) | 0.75 (0.77) | 0.53 (0.58) | 1.42 |
| Dental Materials | 1 (1.11) | 0.83 (0.21) | 1.69 (1.01) | 0.49 |
| Endodontics | 0.65 (0.95) | 0.76 (1.04) | 0.63 (0.83) | 1.21 |
| OMFS | 0.42 (0.82) | 0.4 (0.79) | 0.57 (0.76) | 0.70 |
| Oral Medicine | 0.67 (0.92) | 0.67 (0.68) | 0.68 (1.05) | 0.99 |
| Orthodontics | 0.57 (0.91) | 0.58 (1.13) | 0.54 (0.75) | 1.07 |
| Pathology | 0.67 (1.1) | 0.6 (1.48) | 0.72 (1.08) | 0.83 |
| Pediatric Dentistry | 0.33 (0.58) | 0.63 (0.78) | 0.33 (0.53) | 1.91 |
| Periodontics | 0.38 (1) | 0.58 (1.04) | 0.3 (0.75) | 1.93 |
| Prosthodontics | 0.39 (0.8) | 0.47 (0.72) | 0.35 (0.83) | 1.34 |
| Radiology | 0.5 (0.88) | 0.39 (0.8) | 0.5 (0.98) | 0.78 |
| Restorative Dentistry | 0.33 (0.75) | 0.5 (1.04) | 0.28 (0.67) | 1.79 |

IQR: Inter-Quartile Range; MtoW: Men-to-Women ratio; COH: Community Oral Health; OMFS: Oral and Maxillofacial Surgery; Pathology: Oral and Maxillofacial Pathology; Radiology: Oral and Maxillofacial Radiology.
