# Supplementary material for: An investigation into gender distributions in scholarly publications among dental faculty members in Iran
Source: PLoS One. 2024 Jun 27;19(6):e0300698. doi: 10.1371/journal.pone.0300698 (PMC11210791; doi:10.1371/journal.pone.0300698)
Supplement: Attachment — Submitted filename: review commentsr(Recovered).pdf [file pone.0300698.s009.pdf]

PLOS ONE

An Investigation into Gender Distributions in Scholarly Publications among Dental Faculty Members in Iran

--Manuscript Draft--

**Manuscript Number:**	PONE-D-23-22007
**Article Type:**	Research Article
**Full Title:**	An Investigation into Gender Distributions in Scholarly Publications among Dental Faculty Members in Iran
**Short Title:**	Gender inequality among Iranian dental faculties
**Corresponding Author:**	Ahmad Sofi-Mahmudi, DDS<br>McMaster University<br>Hamilton, Ontario CANADA
**Keywords:**	Gender differences; Scientometric indices; Dental faculties; Iran; Academic Research; Academic Performance
**Abstract:**	Background<br><br>To investigate gender differences in scientometric indices among faculty members in dental schools across Iran. This included overall data and speciality-specific data.<br><br>Methods<br><br>The publication profiles of academic staff in all dental schools were examined using the Iranian Scientometric Information Database (ISID, http://isid.research.ac.ir). Variables analysed were working field, academic degree, total number of papers, papers per year, total number of citations, percentage of self-citation, h-index, g-index, citations per paper, gender, university type, number of years publishing, proportion of international papers, first-author papers, and corresponding-author papers. Mann-Whitney and Kruskal-Wallis nonparametric tests were used to analyse the relationship between background characteristics and scientometric indicators. The extracted data were analysed using R v4.0.1.<br><br>Results<br><br>The database included 1850 faculty members, of which about 60% (1104 of 1850) were women. Men (n=746) had a higher number of papers (6583 vs 6255) and citations (60410 vs 39559) compared with women; 234 of the 376 faculty members with no papers were women. Almost half of the women (N=517 of 1104) were in Type 2 universities, and nearly half of the men (N=361 of the 746) were faculty members at Type 1 universities (Type 1 universities ranking higher than Type 2 and 3 universities). The medians of scientometric indices were higher in men, except for self-citation percentage (0 (IQR=2) vs 0 (IQR=3), P=0.083), international papers percentage (0 (IQR=7.5) vs 0 (IQR=16.7), P<0.001). The proportion of corresponding-author papers was more than 62% higher in women (25 (IQR=50) vs 15.4 (IQR=40), P<0.001). Men had a two-fold higher median h-index (2 (IQR=4) vs 1 (IQR=3), P<0.001). Restorative dentistry and paediatric dentistry had the highest men-to-women ratios (1.5 for both). Dental materials and oral and maxillofacial surgery showed the lowest men-to-women ratios (0.42 and 0.5, respectively).<br><br>Conclusions<br><br>Women made up the majority of dental faculty members in Iran. Nevertheless, men showed better scientometric results in several significant indices. Having identified scientometric information reflecting difference across faculty members, further research is now needed to better understand the drivers of these differences.
**Order of Authors:**	Ahmad Sofi-Mahmudi, DDS
	Erfan Shamsoddin

Powered by Editorial Manager® and ProduXion Manager® from Aries Systems Corporation

	Lisa M DeTora
	Barbara E Bierer
	Perihan Elif Ekmekci
	Morenike Oluwatoyin Folayan
	Ching Shan Lii
	Marcos Roberto Tovani-Palone
	Francis P Crawley
**Additional Information:**	
**Question**	**Response**
**Financial Disclosure**<br><br>Enter a financial disclosure statement that describes the sources of funding for the work included in this submission. Review the submission guidelines for detailed requirements. View published research articles from *PLOS ONE* for specific examples.<br><br>This statement is required for submission and **will appear in the published article** if the submission is accepted. Please make sure it is accurate.<br><br>**Unfunded studies**<br>Enter: *The author(s) received no specific funding for this work.*<br><br>**Funded studies**<br>Enter a statement with the following details:<br>• Initials of the authors who received each award<br>• Grant numbers awarded to each author<br>• The full name of each funder<br>• URL of each funder website<br>• Did the sponsors or funders play any role in the study design, data collection and analysis, decision to publish, or preparation of the manuscript?<br>• **NO** - Include this sentence at the end of your statement: *The funders had no role in study design, data collection and analysis, decision to publish, or preparation of the manuscript.*<br>• **YES** - Specify the role(s) played.<br><br>* typeset	The author(s) received no specific funding for this work.
**Competing Interests**	The authors have declared that no competing interests exist.

Use the instructions below to enter a competing interest statement for this submission. On behalf of all authors, disclose any competing interests that could be perceived to bias this work—acknowledging all financial support and any other relevant financial or non-financial competing interests.<br><br>This statement is required for submission and **will appear in the published article** if the submission is accepted. Please make sure it is accurate and that any funding sources listed in your Funding Information later in the submission form are also declared in your Financial Disclosure statement.<br><br>View published research articles from *PLOS ONE* for specific examples.<br><br>**NO authors have competing interests**<br><br>Enter: *The authors have declared that no competing interests exist.*<br><br>**Authors with competing interests**<br><br>Enter competing interest details beginning with this statement:<br><br>*I have read the journal's policy and the authors of this manuscript have the following competing interests: [insert competing interests here]*<br><br>* typeset	
**Ethics Statement**<br><br>Enter an ethics statement for this submission. This statement is required if the study involved:<br><br>• Human participants<br>• Human specimens or tissue<br>• Vertebrate animals or cephalopods<br>• Vertebrate embryos or tissues<br>• Field research	N/A

Write "N/A" if the submission does not require an ethics statement.

General guidance is provided below. Consult the submission guidelines for detailed instructions. **Make sure that all information entered here is included in the Methods section of the manuscript.**

Format for specific study types

Human Subject Research (involving human participants and/or tissue)

- Give the name of the institutional review board or ethics committee that approved the study
- Include the approval number and/or a statement indicating approval of this research
- Indicate the form of consent obtained (written/oral) or the reason that consent was not obtained (e.g. the data were analyzed anonymously)

Animal Research (involving vertebrate animals, embryos or tissues)

- Provide the name of the Institutional Animal Care and Use Committee (IACUC) or other relevant ethics board that reviewed the study protocol, and indicate whether they approved this research or granted a formal waiver of ethical approval
- Include an approval number if one was obtained
- If the study involved *non-human primates*, add *additional details* about animal welfare and steps taken to ameliorate suffering
- If anesthesia, euthanasia, or any kind of animal sacrifice is part of the study, include briefly which substances and/or methods were applied

Field Research

Include the following details if this study involves the collection of plant, animal, or other materials from a natural setting:

- Field permit number
- Name of the institution or relevant body that granted permission

Powered by Editorial Manager® and ProduXion Manager® from Aries Systems Corporation

**Data Availability**<br><br>Authors are required to make all data underlying the findings described fully available, without restriction, and from the time of publication. PLOS allows rare exceptions to address legal and ethical concerns. See the PLOS Data Policy and FAQ for detailed information.<br><br>A Data Availability Statement describing where the data can be found is required at submission. Your answers to this question constitute the Data Availability Statement and **will be published in the article**, if accepted.<br><br>**Important:** Stating 'data available on request from the author' is not sufficient. If your data are only available upon request, select 'No' for the first question and explain your exceptional situation in the text box.<br><br>Do the authors confirm that all data underlying the findings described in their manuscript are fully available without restriction?	Yes - all data are fully available without restriction
**Describe where the data may be found in full sentences. If you are copying our sample text, replace any instances of XXX with the appropriate details.**<br><br>• If the data are **held or will be held in a public repository**, include URLs, accession numbers or DOIs. If this information will only be available after acceptance, indicate this by ticking the box below. For example: *All XXX files are available from the XXX database (accession number(s) XXX, XXX.*).<br>• If the data are all contained **within the manuscript and/or Supporting Information files**, enter the following: *All relevant data are within the manuscript and its Supporting Information files.*<br>• If neither of these applies but you are able to provide **details of access elsewhere**, with or without limitations, please do so. For example:	The data underlying the results presented in the study are available from our figshare repository at https://doi.org/10.6084/m9.figshare.23669190.

Data cannot be shared publicly because of [XXX]. Data are available from the XXX Institutional Data Access / Ethics Committee (contact via XXX) for researchers who meet the criteria for access to confidential data.<br><br>*The data underlying the results presented in the study are available from (include the name of the third party and contact information or URL).*<br>• This text is appropriate if the data are owned by a third party and authors do not have permission to share the data.<br><br>* typeset	
Additional data availability information:	

Powered by Editorial Manager® and ProduXion Manager® from Aries Systems Corporation

Manuscript

Click here to access/download;Manuscript;Gender Inequality-PLOS.docx

An Investigation into Gender Distributions in Scholarly Publications among Dental Faculty Members in Iran

Short title: Gender inequality among Iranian dental faculties

Ahmad Sofi-Mahmudi[1-3], Erfan Shamsoddin[3], Lisa M DeTora[4], Barbara E Bierer[5], Perihan Elif Ekmekci[6], Morenike Oluwatoyin Folayan[7], Ching Shan Lii[8], Marcos Roberto Tovani-Palone[9], Francis P Crawley[10]

[1] Department of Health Research Methods, Evidence and Impact, McMaster University, Hamilton, Ontario, Canada.

[2] National Pain Centre, Department of Anesthesia, McMaster University, Hamilton, ON, Canada.

[3] Cochrane Iran Associate Centre, National Institute for Medical Research Development (NIMAD), Tehran, Iran.

[4] Department of Writing Studies and Rhetoric, Hofstra University, Hempstead, NY 11549, USA

[5] Division of Global Health Equity, Department of Medicine, Brigham and Women's Hospital, Harvard Medical School, Boston, USA

[6] Department of History of Medicine and Ethics, TOBB ETU University Medical School, Ankara, Turkey

[7] Department of Child Dental Health, Obafemi Awolowo University, Ile-Ife, Nigeria

[8] Department of Pharmacy, Kuala Lumpur General Hospital, Kuala Lumpur, Malaysia

[9] Ribeirão Preto Medical School, University of São Paulo, Ribeirão Preto, Brazil

[10] Good Clinical Practice Alliance-Europe (GCPA) & Strategic Initiative for Developing Capacity in Ethical Review (SIDCER), Leuven, Belgium

Corresponding author: Ahmad Sofi-Mahmudi; **Address**: MDCL-2101, 1280 Main Street West, Hamilton, ON L8S 4K1, Canada; **Telephone**: +1 (905) 525-9140 ext. 22743; **Email**: sofima@mcmaster.ca; a.sofimahmudi@gmail.com.

Abstract

Background: To investigate gender differences in scientometric indices among faculty members in dental schools across Iran. This included overall data and speciality-specific data.

Methods: The publication profiles of academic staff in all dental schools were examined using the Iranian Scientometric Information Database (ISID, http://isid.research.ac.ir). Variables analysed were working field, academic degree, total number of papers, papers per year, total number of citations, percentage of self-citation, h-index, g-index, citations per paper, gender, university type, number of years publishing, proportion of international papers, first-author papers, and corresponding-author papers. Mann-Whitney and Kruskal-Wallis nonparametric tests were used to analyse the relationship between background characteristics and scientometric indicators. The extracted data were analysed using R v4.0.1.

Results: The database included 1850 faculty members, of which about 60% (1104 of 1850) were women. Men (n=746) had a higher number of papers (6583 vs 6255) and citations (60410 vs 39559) compared with women; 234 of the 376 faculty members with no papers were women. Almost half of the women (N=517 of 1104) were in Type 2 universities, and nearly half of the men (N=361 of the 746) were faculty members at Type 1 universities (Type 1 universities ranking higher than Type 2 and 3 universities). The medians of scientometric indices were higher in men, except for self-citation percentage (0 (IQR=2) vs 0 (IQR=3), P=0.083), international papers percentage (0 (IQR=7.5) vs 0 (IQR=16.7), P<0.001). The proportion of corresponding-

author papers was more than 62% higher in women (25 (IQR=50) vs 15.4 (IQR=40), $P<0.001$). Men had a two-fold higher median h-index (2 (IQR=4) vs 1 (IQR=3), $P<0.001$). Restorative dentistry and paediatric dentistry had the highest men-to-women ratios (1.5 for both). Dental materials and oral and maxillofacial surgery showed the lowest men-to-women ratios (0.42 and 0.5, respectively).

Conclusions: Women made up the majority of dental faculty members in Iran. Nevertheless, men showed better scientometric results in several significant indices. Having identified scientometric information reflecting difference across faculty members, further research is now needed to better understand the drivers of these differences.

Keywords: Gender differences, Scientometric indices, Dental faculties, Iran, Academic Research, Academic Performance

Background

In recent decades, gender equality (as a part of social equality) has been increasingly sought globally in the medical sciences. The United Nations links greater gender equality with economic development and prosperity as well as being responsible for a significant share of new economic growth over the past fifty years attributable to greater educational access among girls (1, 2). Gender equality is also seen as a contributing factor to sustainable development (3). Science and technology fields have been less successful in achieving gains toward gender equality than found in other professional areas (4). Skewed application processes, poor mentorship opportunities, and social pressures (such as disproportionate household responsibilities) are suggested to contribute to this problem (4-8). Despite the higher rate of female undergraduate and graduate students in many countries (9), there are fewer female PhD candidates, postdocs, and professors (6, 10). Even for women who gain entry to the sciences, barriers may exist to achieving scientific publications, a particularly important measure for career progression (1, 11).

In dentistry, disparities between men and women researchers in both developed (12, 13) and developing countries (14, 15) have been show to exist, favouring men for funding and other financial support (7, 16). Men submit more research applications and receive more awards than women (17), hold more leadership positions in research projects (18), enjoy higher academic ~~tiers~~ (18, 19), and are assigned more positions on dental journal editorial boards (20). A citation gender gap is also observable (21). Among the studies that have assessed the impact of programs and

projects focused on gender inequality in various countries (5) and professions (19, 22), none has yet attempted to evaluate the gender differences across Iranian dental faculties.

Iran has enjoyed a substantial increase in women's enrolment for higher education, with a significant increase in female graduates with higher education levels (23, 24). During the last decade, the number of female college graduates has increased substantially, leading to a higher percentage of female graduates than males (25). However, men continue to occupy a higher number of editorial and administrative positions (26, 27). According to a previous national analysis on the research performance of Iranian medical academics in 2018, men had a higher median of citations per paper and papers per individual compared to women (28). In Iran scientometric studies ~~accessing~~ gender ratios across dental schools within the country had not previously been undertaken. This study was carried out in 2021 in order to identify possible existing gender publication differences among dental faculty members based on scientometric indices. It includes speciality-specific differences as well across dental schools as identified by the data up to 8 August 2020.

Methods

This was an observational study that reviewed the scientometric data of dental faculty members in Iran in 2020. We accessed Scopus outputs from the Iranian Scientometric Information Database (ISID, http://isid.research.ac.ir), Iran's Ministry

of Health and Medical Education (MOHME) database that aims to aggregate the most up-to-date scientometric data of medical faculty members in Iran (28, 29).

To have a more precise perspective on publication productivity, we excluded from the indices papers with a high number of authors (≥100 authors, e.g. Global Burden of Disease studies) using a specific filter ("filter out high-author articles") on the ISID website.

Variables

We assessed the following ISID variables: 1) Background characteristics: name, affiliation (dental school), university type, speciality, and academic ~~tier~~; 2) Scientometric indicators: total number of papers, total number of citations, percentage of self-citation, h-index, g-index, citation per paper, number of papers in which the researcher was the first author, number of papers in which the researcher was the corresponding author, number of papers in which the researcher collaborated with international researchers, and year of the first published Scopus-indexed paper.

We calculated and added six other variables: gender (male or female; manually done based on name; in cases of doubt, we contacted a student in that dental school to provide more information), university type (1 to 3[1]; in which a lower figure indicates a higher-ranking institution, as determined by Iran's MOHME), number of years

[1] While considering all three types of universities in our analyses, the results of this study includes only Type 1 and Type 2 universities. Type 3 universities are young universities with generally less than 10 years of existence. Most dental faculty members are located in Type 1 and Type 2 universities.

publishing (subtraction of the year of the first published Scopus-indexed paper from 2021), percentage of international papers (number of papers in which the researcher collaborated with international researchers divided by their total number of papers), percentage of first-author papers (number of papers in which the researcher was the first author divided by their total number of papers), percentage of corresponding-author papers (number of papers in which the researcher was the corresponding author divided by the total number of papers), and number of papers per year (number of papers divided by the number of years publishing).

Data extraction and analyses

Data were extracted into a preformatted spreadsheet on 8 August 2020 by AS-M and then cleaned and verified by another author (AM). Descriptive statistics were used to report the mean, median, standard deviation (SD), and range of selected variables. A men-to-women (MtoW) ratio was defined as the mean of each variable for men divided by the same mean for women.

Descriptive statistics for all the scientometric indices were reported using the median and inter-quartile range (IQR) in men and women for each speciality, dental school, academic tier, and university type. As our data were not normally distributed (confirmed by using the Shapiro–Wilk test), Kruskal Wallis nonparametric test was used to examine the relationship between the background characteristics and scientometric indicators. The extracted data were analysed using R v4.0.1 (2020-06-06) (R Core Team, R Foundation for Statistical Computing, Vienna, Austria.

http://www.r-project.org). A No p-value level was considered statistically significant according to the statement by the American Statistical Association (30).

Results

~~Overall perspective~~

~~Almost half of the female dental faculty members (N=517) were in Type 2 universities, while nearly half of the male dental faculty members (48.4%; N=361) were affiliated with Type 1 universities. Figure 1 shows the MtoW ratio of various dental faculty in Iran.~~

[Insert Figure 1 here]

Figure 1. Men-to-women (MtoW) ratio of dental faculties in different universities of medical sciences in Iran.

Type: University type; MtoW: Male-to-female ratio; Misc: Miscellaneous.

A total of 1850 dental faculty members appeared having publications in the ISID database, of which 1104 were women. Men had more papers (6583 vs 6255) and citations (60410 vs 39559). Among the 376 faculty members with ~~zero papers~~, 234 (62.2%) were women. Almost half of women (N=517) were in Type 2 universities, while nearly half of men (48.4%; N=361) were affiliated with Type 1 universities. Figure 1 shows the MtoW ratio of the various dental faculties in Iran.

The median of ~~most~~ scientometric indices was higher in men than women (Table 1), except for the self-citation percentage, the international paper percentage, and the percentage of corresponding-author papers. In these three indices, women showed higher medians (P<0.001). ~~Table 1 shows comparisons for a broader set of indices providing greater detail for both genders.~~

Table 1. Scietometric indices for each gender (median [inter-quartile range])

Index	Both genders	Men	Women	P-value
**Papers**	3 (7)	3 (9)	2 (6)	< 0.001
**Citations**	8 (40.75)	13 (27.2)	6 (63)	< 0.001
**Self-citations (%)**	0 (3)	0 (3)	0 (2)	0.083
**h-index**	1 (3)	2 (4)	1 (3)	< 0.001
**g-index**	2 (5)	2.5 (7)	1 (4)	< 0.001
**Citations per paper**	4 (5.70)	5 (5.93)	3.33 (5.42)	< 0.001
**Years publishing**	8 (6)	8 (7)	7 (5)	< 0.001
**International papers (%)**	0 (11.8)	0 (16.7)	0 (7.5)	< 0.001
**First author papers (%)**	20 (50)	24.5 (46.2)	17.6 (50)	0.122

**Corresponding-author papers (%)**	22.2 (50)	15.4 (40)	25 (50)	< 0.001
**Number of papers per year**	0.5 (0.83)	0.5 (0.83)	0.4 (0.86)	0.006

Gender differences in academic tiers

Women in each academic tier had a higher total number of papers with fewer total citations. The data showed more publication activity, citations, and international collaboration among the higher ranking academic members for both genders (Table 2).

When comparing MtoW ratio between different academic tiers, from assistant professor to full professor, a decreasing trend was seen in the number of citations, h-index, citations per paper, and the number of papers per year. In contrast, the number of papers, working years, and corresponding-author papers percentage increased for women. The highest and lowest MtoW ratio belonged to the self-citation percentage of associate professors (2.00) and corresponding-author papers percentage of assistant professors (0). Full details are illustrated in Appendix Table 1. Rates of first-authored papers and corresponding-author papers tended to favour women in all tiers over men. There were slight differences between genders in years publishing and the number of papers published per year. The complete set of results for these factors is presented in Table 2.

Table 2. Gender publication ratios across academic tiers (median [inter-quartile range])

Index	Assistant professors			Associate professor			Full professors			P-values
	Both	Men	Women	Both	Men	Women	Both	Men	Women	
**Papers**	2 (4)	1 (4)	2 (4)	12 (10)	11 (11)	12 (9.5)	23 (22)	21 (22)	25.5 (21.8)	0.877, 0.873, 0.482
**Citations**	3 (16)	4 (20)	3 (14)	61.5 (90.75)	71 (100)	54 (81.5)	199 (193)	200 (190)	194 (215)	0.037, 0.191, 0.897
**Self-citations (%)**	0 (0)	0 (0)	0 (0)	1 (5)	2 (5)	1 (5.75)	3 (5)	3 (5)	4 (5)	0.588, 0.927, 0.501
**h-index**	1 (2)	1 (2)	1 (2)	4 (3)	4 (3)	4 (3)	8 (4)	8 (4)	8.5 (4.25)	0.064, 0.383, 0.394
**g-index**	1 (3)	1 (3)	1 (3)	7 (5)	7 (6)	6 (5)	13 (6)	13 (7)	12.5 (6.25)	0.098, 0.247, 0.864
**Citations per paper**	2.8 (5)	3.33 (5.57)	2.5 (4.31)	5.33 (4.9)	5.75 (5.1)	4.88 (4.61)	8.22 (4.66)	8.55 (4.36)	7.45 (4.97)	<0.001, 0.240, 0.24[illegible]
**Working years**	6 (5)	6 (4)	6 (4)	11 (4)	11 (4.25)	11 (4)	14 (4)	14 (3.25)	13 (3)	0.021, 0.887, 0.182
**International papers (%)**	0 (4.762)	0 (12.5)	0 (0)	3.64 (12.5)	5.88 (15.4)	0 (10.8)	7.69 (19.191)	10 (22.3)	6.67 (13.6)	0.018, 0.104, 0.393
**First author papers (%)**	0 (36.842)	0 (36.3)	0 (37.3)	37.5 (32.229)	35.3 (30)	42.5 (37.5)	41.67 (26.708)	40 (20.4)	44.7 (29.2)	0.699, 0.016, 0.058
**Corresponding-author papers (%)**	16.67 (50)	0 (42.9)	25 (50)	25 (33.164)	18.2 (30.8)	30.4 (26.2)	25.78 (25.714)	24 (23.0)	30.9 (31.1)	0.001, <0.001, 0.15[illegible]
**Number of papers per year**	0.33 (0.667)	0.33 (0.667)	0.33 (0.667)	1.08 (0.925)	1 (1.03)	1.17 (0.753)	1.71 (1.26)	1.52 (1.27)	1.82 (1.16)	0.661, 0.768, 0.329

P-values order: assistant professors, associate professors, full professors

Gender differences in university types

Men in Type 1 universities had the highest median in all indices except for the percentage of corresponding-author papers. The differences between genders in self-citation percentage, h-index, g-index, working years, and the number of papers per year were slight.

When comparing MtoW ratio between different university types, from Type 1 to Type 2, a decreasing trend was seen in citations per paper, whereas the number of papers per year increased. The highest and lowest MtoW ratio belonged to citations of Type 2 (2.00) and corresponding-author papers percentage of Type 2 (0). Full details are illustrated in Appendix Table 2.

Women in Type 1 universities had the highest corresponding-author papers percentage (27.3%), followed by women in Type 2 universities (25%) and men in Type 1 universities (18.4%). The complete results of these analyses are found in Table 3.

Table 3. Gender differences based on university types (median [inter-quartile range])

**Index**	**Type 1 universities**			**Type 2 universities**			**Type 3 universities**			**P-values**
	Both	Men	Women	Both	Men	Women	Both	Men	Women	
**Papers**	7 (12)	8 (13)	6 (11)	2 (4)	1 (5)	2 (4)	1 (2)	1 (2)	1 (2)	0.877, 0.873, 0.482

**Citations**	28 (89)	39 (118)	23 (70.8)	3 (17)	4 (25)	2 (14)	0 (9)	0 (11)	0 (9)	0.037, 0.191, 0.897
**Self-citations (%)**	1 (4)	1 (4)	0 (4)	0 (0)	0 (1)	0 (0)	0 (0)	0 (0)	0 (0)	0.588, 0.927, 0.501
**h-index**	3 (4)	3 (5)	2 (4)	1 (2)	1 (2)	1 (2)	0 (1)	0 (1.5)	0 (1)	0.064, 0.383, 0.394
**g-index**	4 (8)	5 (8)	4 (7)	1 (3)	1 (3)	1 (3)	0 (2)	0 (2)	0 (2)	0.098, 0.247, 0.864
**Citations per paper**	5.13 (5.83)	6 (5.61)	4.45 (5.63)	2.69 (4.75)	3.3 (5.75)	2.4 (4.4)	3 (5.9375)	3.19 (5.84)	2.88 (5.88)	<0.001, 0.240, 0.248
**Years Publishing**	10 (7)	10 (7)	9 (6)	6 (5)	6.5 (4.25)	6 (5)	5 (4)	6 (4.75)	5 (4)	0.021, 0.887, 0.182
**International papers (%)**	0 (16.667)	4.55 (20)	0 (13.1)	0 (0)	0 (5.56)	0 (0)	0 (0)	0 (26.1)	0 (0)	0.018, 0.104, 0.393
**First author papers (%)**	29.41 (43.809)	30.3 (38.9)	26.7 (50)	0 (43.30357)	0 (38.5)	0 (50)	0 (28.24675)	0 (21.5)	0 (28.6)	0.699, 0.016, 0.058
**Corresponding-author papers (%)**	23.81 (42.857)	18.4 (39.0)	27.3 (47.5)	20 (50)	10.7 (50)	25 (50)	0 (50)	0 (33.3)	0 (50)	0.001, <0.001, 0.151
**Number of papers per year**	0.77 (1.038)	0.75 (1.04)	0.778 (1)	0.33 (0.727)	0.333 (0.8)	0.333 (0.667)	0.2 (0.5)	0.2 (0.5)	0.2 (0.5)	0.661, 0.768, 0.329

P-values order: Type 1 university, Type 2 university, Type 3 university

Gender differences in various dental schools

The national MtoW ratio among dental faculty members was 0.68 in Iran. Five dental schools in Iran with the highest MtoW ratios were the following: Artesh (3.25), Shahed (1.5), Tabriz (1.30), Mashhad (1.14), and Shahid Beheshti (1.05). On the other hand, Alborz (0.22), Qazvin (0.22), Semnan (0.26), Birjand (0.35), and Zahedan (0.35) dental schools had the lowest MtoW ratios.

Gender differences in specialities

The top five faculty members with the highest h-index, number of papers, and citations in endodontics and oral and maxillofacial surgery (OMFS) were all men. In contrast, the top faculty members in community oral health (COH), dental materials, oral medicine, orthodontics, oral and maxillofacial pathology (from now on: pathology), and restorative dentistry were women.

The MtoW ratio in the median h-index was lower than 1 in dental materials (0.42) and OMFS (0.5), whereas this ratio was equal to 2 in paediatric dentistry, periodontics, and orthodontics. The MtoW ratio in median corresponding authorship percentage was lower than 1 in most fields, except for COH (1.13) and dental materials (1.13). The lowest ratios were seen in radiology (0), restorative dentistry (0), and pathology (0.3). The MtoW ratios in the median number of papers per year were highest in periodontics (1.93), paediatric dentistry (1.91), and restorative dentistry (1.79), whereas the ratios were lowest in dental materials (0.49), OMFS (0.70) and radiology (0.78). Other details regarding gender differences in specialities are depicted in Table 4 and Appendix Tables 3-9.

Table 4. Gender differences in each field (median [inter-quartile range])

Field	h-index			Corresponding author %			Papers per year			MtoW
	Both	Men	Women	Both	Men	Women	Both	Men	Women	
**COH**	3 (3.25)	4 (2.5)	3 (3)	33.33 (57.14)	35 (37.7)	30.95 (50)	0.55 (0.61)	0.75 (0.77)	0.53 (0.58)	1.33, 1.13, 1.42

**Dental Materials**	5 (8.75)	5 (0.5)	12 (10.5)	23.61 (21.5)	25 (8.64)	22.22 (31.46)	1 (1.11)	0.83 (0.21)	1.69 (1.01)	0.42, 1.13, 0.49
**Endodontics**	2 (4)	3 (5.8)	2 (4)	21.83 (47.03)	14.84 (33.33)	29.81 (45.31)	0.65 (0.95)	0.76 (1.04)	0.63 (0.83)	1.5, 0.5, 1.21
**OMFS**	1 (3)	1 (4)	2 (2.5)	10.24 (40)	8.7 (41.18)	12.5 (33.33)	0.42 (0.82)	0.4 (0.79)	0.57 (0.76)	0.5, 0.7, 0.7
**Oral Medicine**	2 (3)	2 (2.5)	2 (3)	23.81 (50)	23.81 (38.18)	24.26 (50)	0.67 (0.92)	0.67 (0.68)	0.68 (1.05)	1, 0.98, 0.99
**Orthodontics**	1 (3)	2 (3)	1 (2)	25 (50)	23.3 (42.56)	25 (52.78)	0.57 (0.91)	0.58 (1.13)	0.54 (0.75)	2, 0.93, 1.07
**Pathology**	2 (4)	3 (4)	2 (4)	32.13 (50)	10 (34.29)	33.33 (56.25)	0.67 (1.1)	0.6 (1.48)	0.72 (1.08)	1.5, 0.3, 0.83
**Pediatric Dentistry**	1 (2)	2 (3.25)	1 (2)	25 (50)	16.23 (46.15)	31.58 (50)	0.33 (0.58)	0.63 (0.78)	0.33 (0.53)	2, 0.51, 1.91
**Periodontics**	1 (3)	2 (4)	1 (3)	23.08 (50)	20 (50)	25 (50)	0.38 (1)	0.58 (1.04)	0.3 (0.75)	2, 0.8, 1.93
**Prosthodontics**	1 (2)	1 (2)	1 (2)	17.42 (50)	12.5 (50)	25 (50)	0.39 (0.8)	0.47 (0.72)	0.35 (0.83)	1, 0.5, 1.34
**Radiology**	1 (3)	1.5 (3)	1 (3)	14.29 (50)	0 (25.57)	20 (50)	0.5 (0.88)	0.39 (0.8)	0.5 (0.98)	1.5, 0, 0.78
**Restorative Dentistry**	1 (2.25)	1 (4)	1 (2)	16.03 (50)	0 (20)	25 (50)	0.33 (0.75)	0.5 (1.04)	0.28 (0.67)	1, 0, 1.79

MtoW: Men-to-Women ratio; COH: Community Oral Health; OMFS: Oral and Maxillofacial Surgery; Pathology: Oral and Maxillofacial Pathology; Radiology: Oral and Maxillofacial Radiology.
MtoW order: h-index, corrsponding author %, papers per year.

Discussion

Overall, we found multiple cases of gender differences regarding publication performance among dental faculty members in Iran. The number of female dental faculty members was approximately 48% higher than that of males. Additionally, women had a higher median percentage of corresponding-author papers (15.4 vs 25). These findings appear to show a positive step towards more balanced publication contributions by men and women dental faculty members in Iran. They may also indicate a continued disproportionate assignment of labour for women in conducting dental research. Hence, these findings alone do not indicate either a clear favouring or disfavoring of women compared to men in the dental sciences in Iran. More research is needed to ~~to~~ identify overall trends and underlying causes for ongoing gender differences in scientometric indices among dental faculty members in Iran.

In contrast to some studies indicating unequal academic job opportunities for men and women (18, 31, 32), the opposite appears to have occurred in Iran for dentistry. In Iran, we have seen a strong openness to hiring women as dental faculty members, indicated by the fact that now a significantly higher percentage of new hirings are women as compared to men. This may be an example of the "Gender-Equality Paradox in Science, Technology, Engineering, and Mathematics Education", suggesting that women in countries with a higher rate of gender discrimination are willing to pursue the most straightforward way to financial independence. This trajectory usually leads through science, technology, engineering, and mathematics education and professions (34).

We found that men had a higher mean and median number of working years as researchers (nearly one year of difference). A likely factor contributing to this difference might be their type of working universities as well as developing trends in hiring. Approximately half of the men were affiliated with Type 1 universities, while women mainly were in Type 2 universities. Given that Type 1 universities usually require their tenured members to have a significant research background (including years publishing), the higher number of women in Type 2 universities appears to indicate an employment pattern for dentistry faculty members in which women have generally occupied roles that are considered as "early-career." When researching the number of dental faculty members, the distribution of male to female members noticeably differed among dental schools in Iran. While the national MtoW ratio was favourable (0.68) for women, more ~~gender-aware~~ policies are necessary to move towards gender equality in hiring academic staff ~~in~~ Iranian medical universities. Of course, this requires an existing set of infrastructure to build upon. For instance, Artesh University of Medical Sciences is affiliated with the military forces in Iran. It is thus not surprising to see in that university the highest MtoW ratio in its faculty members.

Women had more corresponding-author papers in all three academic tiers (assistant, associate, or full professor) than men in the same positions. In Iran, the published theses of masters and doctoral students in dental schools tend to assign supervisors as the corresponding authors. This may account for the high prevalence of female corresponding-author papers: female dental faculty members may tend to focus their research projects on supporting the education of their students. Additionally, the

higher research labour that comes from these corresponding roles can limit their available time to engage in international collaborations because the thesis projects are generally conducted on a subnational level and tend to focus on local issues. Our findings appear to substantiate this premise. Women were less likely to take part in international collaborations leading to publication, and men had a higher mean percentage of international papers. This could imply better international partnerships for men (12.30 vs 9.57). These results align with previous studies indicating how gender disparities can occur in scholarly authorship (33).

In the "Global Gender Gap Report 2020" published by the World Economic Forum, among the 153 evaluated countries, Iran was ranked as 148 (down from 142 in 2018) (34). Although there have been enormous efforts in Iran over the past decades to advance women's participation in science (35), men still occupy the highest-ranking administrative positions in the top-ranked dental schools and in MOHME supported institutions generally. According to the ISID platform, less than 20% of dental schools deans in Iran were women (~~16.7%~~, 7 of 42) at the time of our study. We also found that less than one-third (7 of 22) of chief editorial positions among Iran's dental journals were occupied by women (data derived from the MOHME site journals.research.ac.ir). An earlier study noted similar gender differences among dental school faculty in the United States (36).

Female physicians in academia in Iran, on average, receive a lower salary compared with their male counterparts. A similar pattern is found in dentistry at all academic tiers (37, 38). Women faculty members ~~also face~~ are also less represented in awarded grant applications for research funding (39), unequal departmental resource

allocation (40), and difficulty with obtaining the desired mentorship (41). Women may also be disproportionately impacted by personal factors related to societal demands, such as greater responsibilities within the home (42). A less positive work ambience for women in comparison with men in dental schools may also contribute to the differences in publication results, for example, receiving less respect from students, different expectations from patients in clinical procedures, and facing more difficulties in obtaining a work-life balance (43-45). All these factors could reduce the publication productivity of female faculty members. In our study, men were more productive, publishing nearly 55% more papers than women. However, the difference narrows to 26% when considering annual productivity. One large study found that while, on average male scientists publish 13.2 papers during their career, female authors publish only an average of 9.6 throughout their career, resulting in a 27% gender gap in total productivity (32). This is half the gap found in our study, which may suggest that women in Iran are not attracted to dental science production as much as in other parts of the world due to some structural barriers (e.g., their role in family support or potential discriminations they may face in the academic situation in Iran).

The OMFS and prosthodontics field specialities had the highest gender disparities regarding the number of women employed in those faculties. The number of female dental faculty members were found to be highest in the pediatric dentistry speciality. The underrepresentation of women in OMFS (46, 47) and their overrepresentation in pediatric dentistry (48) have been reported in other countries previously. One possible factor causing a notable gender gap in the OMFS field is that OMFS residency

in Iran poses considerable stress and labour burden on residents. Moreover, the duration of speciality education conflicts with childbearing years and might be a potential reason for the specialist gender gap. The higher number of male dental faculty members in this field in the past may be self-perpetuating. On the other hand, pediatric dentistry appears to show less hidden or overt bias and greater acceptability for women (49).

Our research results show promise for an increasing amount of scholarly publication by women in dental faculties in Iran. The trend looks to be new and still faces opposition from a general secular trend in the country. Sustainable changes can only be expected when barriers to the full participation of women in the academic enterprise are reduced (38). With a higher number of female dental faculty members in Iran, it is expected to see an ongoing increase in the number of scholarly publications by female dental faculty members. This is likely to be accompanied by a more general trend toward more opportunities for women to participate in leadership positions in administrative and editorial positions in the future of Iran's dental sciences. ~~Increasing~~ gender equality in dental publications in Iran contributes to an overall global goal of gender equality as a fundamental goal of education, science, and equity in society (50).

Limitations

Our focus on scholarly publication data limits our analysis to appreciating gender differences within the single career measure of publication contributions. It does not take into account other career characteristics, including the career dynamics in which

dental faculty in Iran participate: the important areas of teaching, administration, and contributions to the life of the faculty and the school. In dentistry, contributions to research on medical devices and pharmaceuticals and patent developments are crucial for the development of the dental sciences within the country and internationally. A further limitation was that the Scopus database only includes English language publications, imposing a significant bias on results for a country where English is not the official language, and faculty members are accustomed to publishing in Persian and other international languages. Further research should provide broader, more needed evidence on the underlying factors influencing publication dispositions, career choices, international collaborations, hiring practices, and other gender trends in Iran's dental faculties.

Conclusions

Whereas women occupy more positions in the dental faculties in Iran, men had an overall better scientometric performance. When categorising based on university type and academic tier, however, the differences were reduced markedly. More research in this field should be encouraged and supported in order to better assess trends and underlying issues affecting gender differences among dental faculty members in Iran.

List of abbreviations

COH: Community oral health

OMFS: Oral and maxillofacial surgery

MOHME: (Iran's) Ministry of Health and Medical Education

Declarations

Ethics approval and consent to participate: Not applicable..

Consent to participate: Not applicable.

Consent for publication: Not applicable.

Availability of data and material: The datasets generated during the current study are available in the figshare repository at https://doi.org/10.6084/m9.figshare.23669190.

Competing interests: The authors of this paper have no conflict of interest to disclose.

Funding: None.

Authors' contributions: AS-M: Conceptualization, investigation, writing the original draft, interpreting the data, and revising the manuscript; ES: Investigation, writing the original draft, interpreting the data, and revising the manuscript; LMD: Writing the original draft, interpreting the data and revising the manuscript; BEB: Writing the original draft, interpreting the data and revising the manuscript; PEE: Writing the original draft and revising the manuscript; MOF: Investigation, writing the original

draft, and revising the manuscript; wrote and revised the manuscript; CSL: Investigation, interpreting the data, and revising the manuscript; MRT-P: Writing the original draft and revising the manuscript; FPC: writing the original draft, interpreting the data, and revising the manuscript. All authors reviewed the manuscript.

Acknowledgments

We wish to thank Dr Vasantha Muthuswamy (President, Forum for Ethics Review Committees in India [FERCI], Mumbai, India) for her valuable comments on our paper.

References

1. Nations U. Leave No One Behind: A Call to Action for Gender Equality and Women's Economic Empowerment. eSocialSciences; 2017.
2. Women U. Facts and figures: Economic empowerment 2018 [Available from: https://www.unwomen.org/en/what-we-do/economic-empowerment/facts-and-figures.
3. Victoria SGo. Gender equality: what is it and why do we need it? Australia2021 [Available from: https://www.vic.gov.au/gender-equality-what-it-and-why-do-we-need-it.
4. Howe-Walsh L, Turnbull S. Barriers to women leaders in academia: tales from science and technology. Stud High Educ. 2016;41(3):415-28.
5. Raj A, Kumra T, Darmstadt GL, Freund KM. Achieving Gender and Social Equality: More Than Gender Parity Is Needed. Academic Medicine. 2019;94(11):1658-64.
6. Herschberg C, Berger L. Academic careers and gender inequality: Leaky pipeline and interrelated phenomena in seven European countries: Trento: University of Trento; 2015.
7. Burns KE, Straus SE, Liu K, Rizvi L, Guyatt G. Gender differences in grant and personnel award funding rates at the Canadian Institutes of Health Research based on research content area: A retrospective analysis. PLoS Med. 2019;16(10):e1002935.
8. Ceci SJ, Williams WM. Understanding current causes of women's underrepresentation in science. Proceedings of the National Academy of Sciences of the United States of America. 2011;108(8):3157-62.
9. OECD. Education at a Glance 20202020.

10. De Angelis G, Grüning B. Gender inequality in precarious academic work: female adjunct professors in Italy. Front Psychol. 2020;4:87.
11. Vuong QH. Breaking barriers in publishing demands a proactive attitude. Nature human behaviour. 2019;3(10):1034.
12. Van den Besselaar P, Sandström U. Gender differences in research performance and its impact on careers: a longitudinal case study. Scientometrics. 2016;106(1):143-62.
13. Abramo G, D'Angelo C, Caprasecca A. Gender differences in research productivity: A bibliometric analysis of the Italian academic system. Scientometrics. 2009;79(3):517-39.
14. Paswan J, Singh VK. Gender and research publishing analyzed through the lenses of discipline, institution types, impact and international collaboration: a case study from India. Scientometrics. 2020:1-19.
15. Dayioğlu M, Türüt-Aşik S. Gender differences in academic performance in a large public university in Turkey. High Educ. 2007;53(2):255-77.
16. Eloy JA, Svider PF, Kovalerchik O, Baredes S, Kalyoussef E, Chandrasekhar SS. Gender differences in successful NIH grant funding in otolaryngology. Otolaryngol Head Neck Surg. 2013;149(1):77-83.
17. Garcia M, Tiano J, Contreras O, Hildebolt C, Horsford J, Stewart D. Trends in academic dentistry and oral health research funding by gender. JDR Clin Trans Res. 2020;5(2):176-84.
18. Tiwari T, Randall C, Cohen L, Holtzmann J, Webster-Cyriaque J, Ajiboye S, et al. Gender inequalities in the dental workforce: global perspectives. Adv Dent Res. 2019;30(3):60-8.
19. Simon L, Candamo F, He P, Karhade DS, Pirooz Y, Spinella MK, et al. Gender differences in academic productivity and advancement among dental school faculty. J Womens Health. 2019;28(10):1350-4.
20. Ioannidou E, Rosania A. Under-representation of women on dental journal editorial boards. PLoS One. 2015;10(1):e0116630.
21. Budrikis Z. Growing citation gender gap. Nature Reviews Physics. 2020;2(7):346-.
22. Sartori LRM, Henzel LT, de Queiroz ABL, Ramos EC, de Oliveira LJC, Chisini LA, et al. Gender inequalities in the dental science: An analysis of high impact publications. Journal of dental education.n/a(n/a).
23. Hoseini M, Saeedi A, Salehi J. Effective Factors on Women's Participation in University Administration: A Logistic Model. Iranian Journal of Women's Studies. 2007;1(3):127-41.
24. Rahbari L. Women in higher education and academia in Iran. Sociology and Anthropology. 2016;4(11):1003-10.
25. Moinifar HS. Higher Education of Women in Iran: Progress or Problem? Int J Womens Res. 2012;1(1):43-60.
26. Rahbari L. Women in higher education and academia in Iran. Sociol Anthropol. 2016;4(11):1003-10.
27. Ezazi S. Women's Role in Sociological Journals. Iranian Journal of Research on Women. 2009;4:86-111.

28. Sadeghi-Bazargani H, Bakhtiary F, Golestani M, Sadeghi-Bazargani Y, Jalilzadeh N, Saadati M. The research performance of Iranian medical academics: a National Analyses. BMC Med Educ. 2019;19(1):1-6.
29. Wolverton Jr RE, Davidson K, Moradi S. Electronic resources for health and medical systems in Iran. J Electron Resour Librariansh. 2019;31(1):41-6.
30. Wasserstein RL, Schirm AL, Lazar NA. Moving to a World Beyond "p < 0.05". Am Stat. 2019;73(sup1):1-19.
31. Overholtzer L, Jalbert CL. A "Leaky" Pipeline and Chilly Climate in Archaeology in Canada. Am Antiq. 2021:1-23.
32. Huang J, Gates AJ, Sinatra R, Barabási AL. Historical comparison of gender inequality in scientific careers across countries and disciplines. Proceedings of the National Academy of Sciences of the United States of America. 2020;117(9):4609-16.
33. West JD, Jacquet J, King MM, Correll SJ, Bergstrom CT. The role of gender in scholarly authorship. PloS ONE. 2013;8(7):e66212.
34. Forum WE. Global Gender Gap Report 2020. Switzerland: World Economic Forum; 2020.
35. Rezaei A. Can self-efficacy and self-confidence explain Iranian female students' academic achievement? Gender and Education. 2012;24(4):393-409.
36. Simon L, Candamo F, He P, Karhade DS, Pirooz Y, Spinella MK, et al. Gender Differences in Academic Productivity and Advancement Among Dental School Faculty. Journal of women's health (2002). 2019;28(10):1350-4.
37. Jena AB, Olenski AR, Blumenthal DM. Sex Differences in Physician Salary in US Public Medical Schools. JAMA internal medicine. 2016;176(9):1294-304.
38. Ioannidou E, D'Souza RN, Macdougall MJ. Gender equity in dental academics: gains and unmet challenges. Journal of dental research. 2014;93(1):5-7.
39. Kaatz A, Lee YG, Potvien A, Magua W, Filut A, Bhattacharya A, et al. Analysis of National Institutes of Health R01 Application Critiques, Impact, and Criteria Scores: Does the Sex of the Principal Investigator Make a Difference? Academic medicine : journal of the Association of American Medical Colleges. 2016;91(8):1080-8.
40. Holliday E, Griffith KA, De Castro R, Stewart A, Ubel P, Jagsi R. Gender differences in resources and negotiation among highly motivated physician-scientists. Journal of general internal medicine. 2015;30(4):401-7.
41. DeCastro R, Griffith KA, Ubel PA, Stewart A, Jagsi R. Mentoring and the career satisfaction of male and female academic medical faculty. Academic medicine : journal of the Association of American Medical Colleges. 2014;89(2):301-11.
42. Ly DP, Jena AB. Sex Differences in Time Spent on Household Activities and Care of Children Among US Physicians, 2003-2016. Mayo Clinic proceedings. 2018;93(10):1484-7.
43. Tiwana KK, Kutcher MJ, Phillips C, Stein M, Oliver J. Gender issues in clinical dental education. Journal of dental education. 2014;78(3):401-10.
44. Smith MK, Dundes L. The implications of gender stereotypes for the dentist-patient relationship. Journal of dental education. 2008;72(5):562-70.

45. Haden NK, Hendricson W, Ranney RR, Vargas A, Cardenas L, Rose W, et al. The quality of dental faculty work-life: report on the 2007 dental school faculty work environment survey. Journal of dental education. 2008;72(5):514-31.
46. Marti KC, Tishko G, Edwards SP, Inglehart MR. Dental students' OMFS-related experiences and interest in OMFS careers: An exploration. Journal of dental education. 2020.
47. Rostami F, Ahmed AE, Best AM, Laskin DM. The changing personal and professional characteristics of women in oral and maxillofacial surgery. Journal of oral and maxillofacial surgery : official journal of the American Association of Oral and Maxillofacial Surgeons. 2010;68(2):381-5.
48. Dhima M, Petropoulos VC, Han RK, Kinnunen T, Wright RF. Dental students' perceptions of dental specialties and factors influencing specialty and career choices. Journal of dental education. 2012;76(5):562-73.
49. Surdu S, Mertz E, Langelier M, Moore J. Dental Workforce Trends: A National Study of Gender Diversity and Practice Patterns. Medical care research and review : MCRR. 2021;78(1_suppl):30s-9s.
50. UNESCO. Education 2030. Incheon Declaration and Framework for Action. Towards Inclusive and Equitable Quality Education and Lifelong Learning for All. 2015.

Figure 1 Click here to access/download;Figure;Figure 1.jpg

Type	School	MtoW	Type	School	MtoW	Type	School	MtoW	Type	School	MtoW
National	National	0.68	Type 2	Urmia	0.86		Golestan	0.52		Bushehr	1.00
Type 1	Tabriz	1.30		Lorestan	0.81		Shahrekord	0.50		Bojnurd	0.61
	Mashhad	1.14		Gilan	0.78		Arak	0.50		Kurdistan	0.47
	Shahid Beheshti	1.05		Kermanshah	0.72		Zanjan	0.48		Ilam	0.44
	Isfahan	0.79		Babol	0.71		Hormozgan	0.47		Qom	0.41
	Ahwaz	0.77		Ardabil	0.70		Zahedan	0.35		Yasuj	0.38
	Tehran	0.63		Yazd	0.69		Birjand	0.35		Alborz	0.22
	Mazandaran	0.56		Hamadan	0.68		Semnan	0.26	Misc	Shahed	1.50
	Shiraz	0.50		Rafsanjan	0.60		Qazvin	0.26			
	Kerman	0.49		Kashan	0.55	Type 3	Artesh	3.25			

Supporting Table 1

Click here to access/download
Supporting Information
Supplementary Table 1.docx

Supporting Table 2

Click here to access/download
Supporting Information
Supplementary Table 2.docx

Supporting Table 3

Click here to access/download
Supporting Information
Supplementary Table 3.docx

Supporting Table 4

Click here to access/download
Supporting Information
Supplementary Table 4.docx

Supporting Table 5

Click here to access/download
Supporting Information
Supplementary Table 5.docx

Supporting Table 6

Click here to access/download
Supporting Information
Supplementary Table 6.docx

Supporting Table 7

Click here to access/download
Supporting Information
Supplementary Table 7.docx
