# Supplementary material for: An investigation into gender distributions in scholarly publications among dental faculty members in Iran
Source: PLoS One. 2024 Jun 27;19(6):e0300698. doi: 10.1371/journal.pone.0300698 (PMC11210791; doi:10.1371/journal.pone.0300698)
Supplement: Attachment — Submitted filename: Response to reviewers_PLOS ONE.docx [file pone.0300698.s008.docx]

Reviewer #1: This paper brings a very important and recent theme. Authors investigated gender differences in several scientometric indices among dental researchers in Iran. The manuscript is well written, results are solid and an appropriate discussion was written. After the reviewing the paper, my comments are listed below:

Introduction
- Very informative and well written. No suggestion for this section.

Authors: Thanks for your kind comments.

Methods
- Do you think that "observation study" is the most appropriate definition for a scientometric study?

Authors: Thanks for your comment. We agree with you. To prevent confusion with the typical definitions of “observational studies,” we changed the study design name to “cross-sectional scientometric.” Now it reads:

“This was a cross-sectional scientometric study that reviewed the data of dental faculty members in Iran in 2020.”

- Did you collect data about research funding and other financial support? How this could have impacted scientometrics?

Authors: Thanks for your comment. The ISID database does not have such kind of data, and there is a reason for that: Dental faculty members in Iran (unlike countries with a free market) very rarely get industrial funding. Based on our experience (ASM and ES) at the national granting body of Iran (NIMAD), they even rarely apply for funding from such institutions. The oral and dental research in Iran is mainly unfunded or funded locally by the university. Therefore, we do not think seeking funding sources will add much value to the study.

Results
- It would be interesting to present names, positions and research area of the most prolific men and women Iranian authors.

Authors: Thanks for your comment. We had already mentioned the top five men in the first sentence of the “Gender differences in specialties” subsection. However, we added the information about the top women, as follows:

“The top women with the highest h-index had an overall rank of 15–20 (dental materials, pathology, orthodontics, and prosthodontics).”

- It would also be relevant if some of these researchers were interviewed to talk about their careers showing what, in their opinion, were the most important aspects that contributed for the position they have achieved.

Authors: We appreciate your comment, and we think this is a very interesting idea. We also have mentioned the next steps in the last sentence of the “Limitations” subsection in the Discussion. We will consider this as our next project about gender inequalities in Iran.

Discussion
Adequate

Authors: Thanks for your kind comment.
