# Supplementary material for: An investigation into gender distributions in scholarly publications among dental faculty members in Iran
Source: PLoS One. 2024 Jun 27;19(6):e0300698. doi: 10.1371/journal.pone.0300698 (PMC11210791; doi:10.1371/journal.pone.0300698)
Supplement: Attachment — Submitted filename: Response to reviewers_PLOS ONE_rev2.docx [file pone.0300698.s010.docx]

Reviewer #2: 1. Abstract

- The background statement is an aim.

- A more suitable introductory paragraph for the background is required.

Authors: Thanks for your comment. We added a background sentence to the abstract.

2. Methods

- Observationl study - should be more specific i.e., cross sectional.

Authors: Thanks. Amended.

- Describe what 'Scopus outputs' are.

Authors: We have already described the Scopus outputs in the variables section. Those included “total number of papers, total number of citations, percentage of self-citation, h-index, g-index, citation per paper, number of papers in which the researcher was the first author, number of papers in which the researcher was the corresponding author, number of papers in which the researcher collaborated with international researchers, and year of the first published Scopus-indexed paper.”

- Variables

* Is the 'name' variable related to the name of the faculty members? If so, does the actual name of the author have any bearing on the analysis and study outcomes?

Authors: The variable name includes the names of the faculty members. We used this variable to detect the gender of the faculties.

* 'Year of the first published Scopus indexed paper' - are papers which are not Scopus indexed not included in the study? If so, should this be stated as an exclusion criteria?

Authors: As we have mentioned in the first paragraph of the Methods, we only used the Scopus data. Therefore, it can be implied from the text that our focus is only on Scopus and not other sources such as Google Scholar.

- Data sourcing and collection steps could be more descriptive - keywords to source dental papers and faculty members; justification for sourcing only Scopus-indexed papers. Were staff categorised into full/part time staff?

Authors: Thanks for the comment. As indicated in the first paragraph of the Methods, the ISID is a formal database of the Ministry of Health of Iran. They have the records of all the faculty members, including those working in dental schools. Regarding using Scopus-indexed papers, it was the only option that we had and that database does not provide the records for other databases. Also, we do not have part-time dental faculties in Iran. They all are full-time.

3. Results

- Overall perspective

* 1st paragraph is repeated in second paragraph, line 4.

Authors: Thanks for the comment. We fixed that problem.

* 2nd paragraph, line 4 : consider rephrasing to the opposite perspective e.g., over half of the women where in type 1 universities. Conversely, over half of the men were in type 2 universities.

Authors: Thanks for your comment. We have three university types in Iran, and therefore, we cannot say that most of the women were affiliated with Type 1 universities. The highest percentage of affiliations for genders are the ones that are mentioned in the text (men: Type 1, women: Type 2).

- Table 2: Is index 'working years' the same as index 'years publishing'? index 'years' publishing' is used in Table 1&3.

Authors: Thanks for your keen comment. We changed “Years publishing” to “Working years.”

- Section 'gender differences in specialities'

* paragraph 1, line 3: Do the top faculty members for COH, dent mat, oral med etc also have the highest 'h-index, number of papers and citations' as described in the previous sentence or is their high performance in other indices? The sentence needs to be more clear.

Authors: Thanks for the comment. We added the criteria (just as described in the previous sentence).

4. Discussion

- paragraph 1, line 9-11 may be better suited as a concluding statement.

Authors: Thanks for your comment. We removed the sentence. The conclusion already had a similar sentence.

- paragraph 2, line 4 "...indicated by the fact that now a significantly higher percentage of new hirings are women as compared to men." requires a reference

Authors: Thanks for your comment. That sentence was based on the results of our study that the number of female faculty members is higher than men. We amended the sentence.

- paragraph 2, line 5-10 : gender equality paradox statement may require rephrasing. The concept speaks more to women in more democratic, equitable and developed countries being less likely to pursue STEM studies and careers. The deduction of the oppposite - women in non-egalitarian / inequatable countries are more likely to pursue careers in STEM - is not necessarily true.

Authors: Thanks for your keen comment. We rephrased the paragraph accordingly.

- parapgrpah 3 line 4-5: the MtoW representation seems to be contradictory to the 1st paragraph in Results. The sentence states that almost 50% (i,e., less than half; 517/1104=47%) of women are in type 2 universities and 48.4% of men are in type 1 universities. This means there are more women (53% (100%-43%)) in type 1 uni's and more men in type 2 uni's. The footnote in the Methods section states that only results of type 1 and 2 universities were considered. Gender distrubution between type 1&2 universities requires clarification.

Authors: Thanks for the comment. As mentioned in a previous comment (and also in the methods), we have three university types in Iran. In the footnote in the Methods we meant we included all the faculty members in the analyses but in the “Gender differences in university types” section, we only reported the results for type 1 and 2 universities. To avoid confusion, we removed the first sentence of the footnote.

- paragraph 3 line 12: consider 'gender mainstreaming' instead of 'gender aware'.

Authors: Thanks for your keen comment. Amended accordingly.

- paragraph 4 line 5 - 9 statement needs a reference/s.

Authors: Thanks for your comment. These statements are only our guesses for explaining “the high prevalence of female corresponding-author papers.” Therefore, we think we do not need any references for these sentences.

- paragrpah 5 - introduce paragraph as an illustration of the possible barriers and challenges that contribute to the gender distribution disparity in scholarly publications.

Authors: Thanks for your comment. We mentioned some of the barriers and challenges at the end of the paragraph.

- paragraph 5, line 9-10 - referencing style inconsistent

Authors: Thanks for your comment. Since it is not a reference rather a website to catch the data, we preferred to mention the web address in the text.

- paragraph 6 line 13 - this may be incorrectly worded. Men did not publish nearly 55% more papers than women. Rather, men published 328 more papers than women, consequently contriubting to nearlyy 55% of the publication output by dental faculties.

Authors: Thanks for your comment. We amended it accordingly.

- paragraph 6 line 13 - should these calculations be accounted for in the results section?

Authors: Thanks for the comments. Since it needs trivial calculations, we suppose that readers can do the calculations themselves.

Typography and grammer

- Desription belown figure 1: MtoW: Male-to-female ratio - change to male-to-women ratio.

Authors: Thanks for your comment. Amended.

replace academic 'tier' with academic 'rank'

Authors: Thanks for your comment. Amended.

- Background paragraph 3, line 9 : assessing instead of accessing

Authors: Thanks for your comment. Amended.

- women instead of female (term consistency)

Authors: Thanks for your comment. Amended.
